# Ethnic Differences in Postacute Stroke Rehabilitation

**DOI:** 10.1016/j.apmr.2025.11.008

**Published:** 2025-11-21

**Authors:** Imadeddin Hijazi, Amanda Malingagio, Emily Anderson, Madeline Kwicklis, Lynda D. Lisabeth, Lewis B. Morgenstern

**Affiliations:** a Stroke Program, Department of Neurology, University of Michigan Medical School, Ann Arbor, MI; b Department of Epidemiology, The University of Michigan School of Public Health, Ann Arbor, MI.

**Keywords:** Hispanic/Latinx, Mexican American, Outcome, Recovery, Rehabilitation, Stroke

## Abstract

**Objective:**

To investigate ethnic differences in poststroke rehabilitation between Mexican American (MA) and non-Hispanic White (NHW) stroke patients.

**Design:**

Prospective cohort study.

**Setting:**

Community-based (December 2019 to August 2024).

**Participants:**

Of 1453 stroke patients (N=1453), the median age was 66 years, 46% were women, and 66% were MA.

**Interventions:**

Not applicable.

**Main Outcome Measures:**

Stroke patients or their proxies were contacted every 2 weeks for 90 days after stroke to identify their rehabilitation provider: inpatient rehabilitation facility (IRF), skilled nursing facility, home health agency (HHA), or outpatient rehabilitation. Binomial regression was used to model the association of ethnicity (MA vs NHW) with IRF as the first rehabilitation provider. Sequential modeling and propensity-score adjustment were used to investigate demographic and clinical variables impacting ethnic disparities.

**Results:**

Rehabilitation services were received by 974 patients in the 90 days after stroke; 34% of patients received no rehabilitation for the first 2 calls. The most common transitions between the first 2 providers were HHA to home without rehabilitation (14%), IRF to HHA (10%), and IRF to home without rehabilitation (7%). NHW patients had higher IRF usage as the first provider compared with MA patients (NHW vs MA risk difference=7.4%, 95% CI, 0.8-14.0). This difference remained after adjustment for patient and clinical factors (risk difference=6.5%, 95% CI, −0.1 to 13.0) and was attenuated by 74.6% after further adjustment for socioeconomic status (risk difference=1.9%, 95% CI, −5.2 to 9.0). Propensity-score methods implemented in a separate model confirmed a smaller, nonsignificant effect (risk difference=2%, 95% CI, −6 to 9) given observed covariates.

**Conclusions:**

MA stroke patients are less likely to receive rehabilitation in an IRF as the first provider after stroke compared with NHW stroke patients. This difference is largely explained by socioeconomic status.

Rehabilitation is an important facet of stroke recovery with multiple care providers including inpatient rehabilitation facilities (IRF), skilled nursing facilities (SNF), outpatient rehabilitation facilities, or home health agency (HHA) services. This range of providers similarly represents a wide range of rehabilitation intensity, with IRF being most intensive.^1^ Intensive rehabilitation is associated with reduced stroke disability.^2,3^ The most recent Guidelines for Adult Stroke Rehabilitation and Recovery from the American Heart/American Stroke Association from 2025 state that qualifying stroke patients should be cared for in an IRF.^4^ IRF is recommended for patients who meet IRF criteria, whereas outpatient/HHA/SNF may be appropriate based on functional presentation and goals. Appropriate candidates are patients expected to make significant enough recovery to return to the community and can participate in at least 3 hours a day of intensive rehabilitation therapy, 5 days per week.^4^ In a small pilot study, it was shown that Mexican Americans (MAs) may be less likely to receive stroke rehabilitation in an IRF than non-Hispanic Whites (NHWs).^5^

Hispanic/Latin(a,e,o,x) (H/L) are a fast-growing minority group in the United States. MAs are the most numerous subgroup of the H/L population in the US^6^ and previously faced a disproportionate risk of stroke,^7^ although the disparity has since narrowed.^8^ As the MA population continues to grow and age, the need for poststroke rehabilitation among MAs will continue to rise. Minimal data on stroke rehabilitation providers or transitions between provider types for MAs exist. We therefore conducted an observational study of postacute care after acute stroke hospitalization as part of the Brain Attack Surveillance in Corpus Christi Postacute Care (BASIC-PAC) project to determine initial and subsequent rehabilitation providers and to investigate whether MAs receive stroke rehabilitation in less intensive settings compared with NHWs.

This paper reports results from a primary, prespecified specific aim from the BASIC-PAC study (NIH R01NS107463).

## Methods

### Setting

Detailed methods for BASIC-PAC study have been published.^9^ The study took place in the biethnic community of Nueces County, Texas, where 63% of its population identifies as MA and 30% identifies as NHW.^10^ There is minimal influx and efflux of residents, meaning patients can be followed from stroke occurrence to beyond hospital discharge.^10^ The county has 7 hospitals, including 1 comprehensive stroke center.

### Standard protocol approvals, registrations, and patient consents

Participants (or proxies for severe stroke patients) provided written informed consent. This project was approved by Institutional Review Boards at The University of Michigan and CHRISTUS Spohn Health System.

### Participant information

Methods used for stroke ascertainment have been previously published.^11^ Briefly, medical records of potential stroke patients aged >45 with a permanent residential zip code in Nueces County were screened and abstracted by trained coordinators. Stroke fellowship trained physicians validated all possible strokes using source documentation blinded to ethnicity and age. All stroke patients were invited to participate in a baseline interview. Their proxy was invited to participate if the patient was unable to communicate. All interview questions were translated into Spanish and interviewers were bilingual. Baseline interviews include questions regarding age, sex, race, ethnicity, education (less than high school, completed high school, more than high school), income (<$30,000, $30,000-$49,999, $50,000+), health insurance (none/Medicaid only, vs all others), marital status, language (English/Spanish), current smoker, and prestroke function (modified Rankin Scale [mRS]^12^), and cognition (Informant Questionnaire on Cognitive Decline in the Elderly^13^). Medicaid only and no insurance were combined because of their relatively small numbers.

Information collected from medical records included demographics, risk factors and comorbidities (hypertension, diabetes mellitus, smoking, alcohol, high cholesterol, body mass index, coronary heart disease, atrial fibrillation, previous stroke/transient ischemic attack (TIA), dementia, heart failure, end-stage renal disease, cancer, and chronic obstructive lung disease), treatment with thrombolysis/thrombectomy, and initial stroke severity measured by the National Institute of Health Stroke Scale (NIHSS).^14^ In-hospital complications that could affect rehabilitation placement were identified by International Classification of Disease-10 code discharge data (acute kidney injury, sepsis, fall, pneumonia, pressure ulcer, and/or urinary tract infection).^15^

The study included stroke patients identified between December 2019 and August 2024, who agreed to participate in the baseline interview, and completed at least 1 postdischarge 2-week call ascertaining their location (see below).

### Outcome assessment

To identify rehabilitation providers, patients and/or family members were called every 2 weeks during the 90-day poststroke follow-up period to determine their location (home vs other setting). Stroke survivors who died in the hospital or were discharged to hospice were not eligible. For patients not residing at home, information on their facility was collected. For patients residing at home, they were asked to identify the type of rehabilitation they were receiving, if any. Study coordinators compared this information with a detailed list of providers to confirm rehabilitation provider at each time point. Although data were gathered regarding rehabilitation providers every 2 weeks, we examined only the first rehabilitation provider and the transition from the first to second provider.

### Statistical analysis

Variable summaries—median and interquartile range for continuous variables, counts and percentages for categorical ones—were reported by ethnic group, and univariate associations between the variables were evaluated with t tests or Fisher exact tests. We conducted a descriptive analysis of the initial rehabilitation provider and the transitions from the first to second rehabilitation provider in the 1453 stroke survivors who completed the baseline interview and at least 2 outcome assessments. Associations between ethnicity and the initial provider, and ethnicity and the transitions from first to second provider, were assessed with Fisher exact tests (limited to transitions with at least 20 observations) and 2-proportion chi-squared tests for individual categories. Bonferroni-Holm multiple-testing correction was applied to account for multiple comparisons.^16^ Transitions from higher intensity to lower intensity providers and vice versa, as defined by Kind et al,^17^ were also explored using similar methods, with the exception that multiple-testing corrections were not applied given the small number of categories.

Among the subset of stroke patients who received any rehabilitation within the 90-day period, missing data were multiply imputed by chained equations, as implemented in R package mice,^18^, which is standard to BASIC data analysis^19^ and clinical and survey data in general.^20,21^ Continuous variables were imputed with predictive mean matching, and categorical variables with logistic and polytomous logistic regression.

Then, within each of the 20 imputed datasets, propensity scores (PS) were calculated. This was done with logistic regression of NHW ethnicity with predictors that included age, sex, initial NIHSS, treatment with thrombolysis/thrombectomy, stroke/transient ischemic attack history, income, education, insurance status, prestroke disability measured by mRS and cognition (Informant Questionnaire on Cognitive Decline in the Elderly), stroke risk factors, and number of comorbidities. The resulting predicted probabilities of NHW ethnicity were incorporated as PS in the IRF models. PS effectively balance the distribution of specified potential confounders between the exposure groups, in this case the 2 ethnic groups.

Logistic regression was deemed inappropriate, given the high proportion of IRF users which would reduce the interpretability of the odds ratio.^22–24^ Instead, binomial regression with an identity link, which in contrast to logistic regression fits percentage-point differences rather than log-odds ratios, was used to estimate the association between IRF as the first rehabilitation provider and ethnicity. The model was adjusted for the ethnic PS to determine if any ethnic effect was independent of the existing covariates. The relationship between IRF usage and PS score was flexibly estimated, using cubic splines with 4 knots and a smoothing penalty, implemented with R package mgcv.^25^ Higher numbers of knots did not improve model fit by Akaike Information Criterion and deviance residual diagnostics. Predicted values of the observations were confirmed to fall within the (0, 1) interval. The main model also adjusted for hospital system. Results were pooled across imputed datasets using Rubin’s rules. The unadjusted association was also reported. Prespecified analyses examined possible differences in ethnic disparities in IRF usage by education and income, with separate models that included interactions between each socioeconomic variable and ethnicity.

Four sensitivity analyses were performed. The first examined the potential role of sample selection bias by repeating the main analysis with the addition of inverse probability weighting. Medical record data were available for nonparticipants up to September 2023 (780 of 974). The analysis was restricted to this time frame. Weights were computed as the inverse of the predicted values generated by logistic regression of baseline participation status, with the medical record variables as predictors, and incorporated into the PS-adjusted model.

The second sensitivity analysis assessed the robustness of the main PS model by adjusting for the most important covariates in addition to the PS in the main model, including income, insurance status, stroke severity, and prestroke cognition and function. The third included complications that demonstrated a suggestive association with ethnicity (P<.20; falls and pneumonia) in univariate analyses in the calculation of the PS to determine if the main results were robust to different rates of complications between ethnic groups. In the last sensitivity analysis, the main analysis was repeated except the outcome was IRF usage at any time point rather than IRF as the first provider.

In additional post-hoc analyses, to explore which covariates potentially contributed to the observed unadjusted ethnic difference in IRF usage that was mitigated in the PS-adjusted analysis, individual covariates were each added to the model including only ethnicity and these adjusted effects as well as percent change in the ethnicity coefficient from the unadjusted model were reported. Then, hierarchical regression models were run; 6 models were specified. Model 0 was unadjusted. Model 1 adjusted for demographics (age, sex). Model 2 added treatment with thrombolysis/thrombectomy, initial NIHSS, and prestroke disability and cognition to the adjustment. Model 3 added adjustment for comorbidity burden. Model 4 further adjusted for socioeconomic factors (education and income). Model 5 added the hospital system to the adjustment.

## Results

Of the 1453 patients with at least 2 calls, 25% initially went to IRF; this included 24% of MAs and 27% of NHWs. Of the 974 who received any rehabilitation, 38% went to IRF; this included 36% of MAs and 43% of NHWs. Of the 398 rehab recipients with NIHSS of 6 or more, 47% went to IRF; this included 44% of MAs and 55% of NHWs.

Demographic information on the study sample of 1453 stroke survivors is presented in table 1. The median age was 66 years, 46% were women, and 66% were MA. Survival was high in this sample; 98% survived the 90-day period, and among those who died, a median survived 67 (48-74) days.

Of the 974 participants who received any rehabilitation within 90 days, 660 identified as MA (68%), and 314 identified as NHW (32.2%). The median age was 69 years and 49% identified as women (table 1). MA patients had more severe prestroke disability (mRS 4-5; MA=16%; NHW=10%; *P*=.041), were younger (median ages: MA=68; NHW=73; *P*<.001), had lower socioeconomic status (SES) as measured by income and educational attainment (*P*<.001), were more likely to have Medicaid only or be uninsured (MA=19%, NHW=9%, *P*<.001), and had a lower prevalence of thrombolysis/thrombectomy (MA=19%; NHW=26%; *P*=.009). Similar patterns of ethnic differences for insurance status, treatment, age, and SES were observed among IRF recipients (supplemental table S1, available online only at http://www.archives-pmr.org/). Among the 974, 97% survived the 90-day period, and among those who did not survive, the median number of days survived was 60 (39-71).

**Fig 1:**
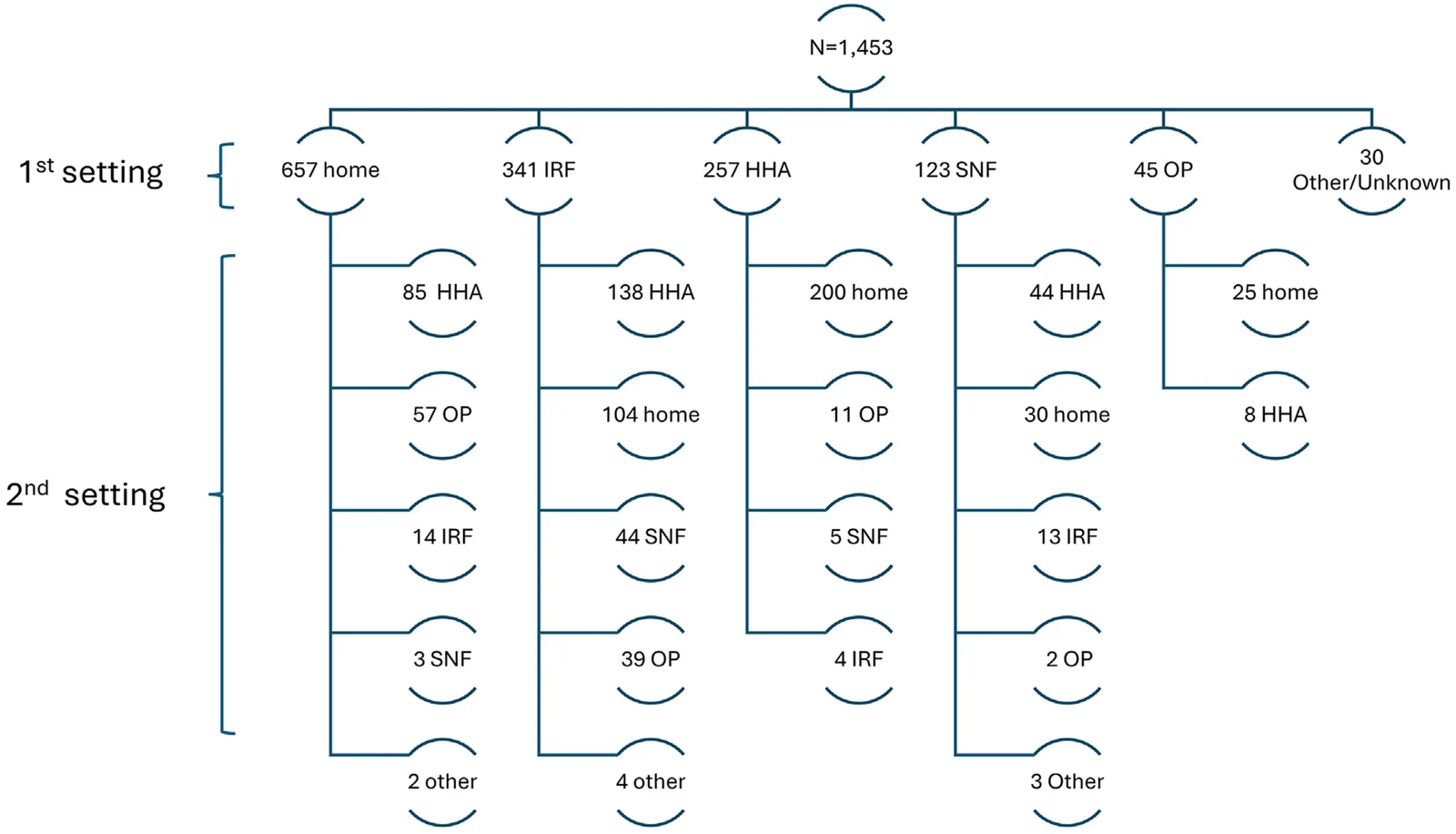
First 2 rehabilitation providers. “Home” refers to home without rehabilitation. HHA, home health agency; IRF, inpatient rehabilitation; OP, outpatient rehabilitation; SNF, skilled nursing facility.

Table 2 shows the initial rehabilitation providers. Of the 974 patients who received any rehabilitation, 36% of MAs and 43% of NHWs went to IRF as their first rehabilitation setting. Among the 1,453 with at least 2 calls, the most common transitions between the first 2 providers were HHA to home without rehabilitation (14%), IRF to HHA (10%), and IRF to home without rehabilitation (7%) (fig. 1). Overall, there was an ethnic difference in these transition patterns (*P*<.001; table 3). NHWs were more likely to transition from IRF to SNF (5% NHW vs 2% MA, adjusted *P*=.032), and MAs were more likely to transition from HHA to home without rehabilitation (10% NHW vs 16% MA, adjusted *P*=.029). Likewise, patterns in the transition intensity also differed by ethnicity (*P*<.001); NHWs were more likely to remain at the same rehabilitation intensity for the first 2 calls (55% NHW vs 45% MA), whereas MAs were more likely to have a complex transition from a lower to higher intensity rehabilitation type (13% MA vs 10% NHW), and a transition from a higher to lower intensity rehabilitation type (43% MA vs 36% NHW) (supplemental table S2, available online only at http://www.archives-pmr.org/).

In the unadjusted analysis of the 974 rehab participants, the proportion of NHW patients participating in IRF as the first rehabilitation provider was 7.4 percentage points higher compared with MA patients (95% CI, 0.8-14.0; *P*=.028) (table 3). After PS adjustment, this difference was attenuated to 1.6% and was no longer statistically significant (95% CI, −6.1 to 9.2; *P*=.687) (supplemental table S3, available online only at http://www.archives-pmr.org/). This result was consistent across various sensitivity analyses, including adjustment for additional demographic and clinical variables in the main model (risk difference=1.5%; 95% CI, −5.9 to 8.8, *P*=.694) along with inclusion of poststroke complications in the PS model (risk difference=1.1%; 95% CI, −6.6 to 8.8; *P*=.779; supplemental table S4, available online only at http://www.archives-pmr.org/), and weighting for baseline attrition (risk difference=4.1 percentage points, 95% CI, −6.1 to 9.2; *P*=.356; supplemental table S5, available online only at http://www.archives-pmr.org/). In addition, the results for any IRF (adjusted percentage points difference=1.6, 95% CI, −6.1 to 9.4) were extremely similar to the results of IRF as the first provider.

Examination of the ethnicity association by income and education indicated potentially meaningful heterogeneity by socioeconomic groups, but the interaction terms were not significant (supplemental table S6, available online only at http://www.archives-pmr.org/).

Subsequent analyses suggested that most of the reduction in the unadjusted vs adjusted ethnicity association was driven by the inclusion of socioeconomic factors in the model (74.6% change, P=.605), with education alone accounting for a roughly 55% reduction in the effect size of ethnicity with no further adjustment (table 4).

## Discussion

Our analysis showed that MA stroke patients were less likely to be admitted to an IRF as the initial rehabilitation setting after stroke compared with NHW stroke patients because of their lower SES (income and education). The MA:NHW ethnicity difference was not statistically significant after adjustment, and the attenuation was mainly because of SES. It has been consistently reported that stroke patients whose care included an IRF make more functional gains,^26,27^ so this finding is discouraging. Recent literature has indeed shown a negative relationship between SES and rehabilitation intensity.^28^ This discrepancy is difficult to explain given that insurance is often the main payer for IRF as opposed to out-of-pocket expenses. In our sample, MAs were more likely to be uninsured and to have Medicaid than NHWs, but insurance status had far less of an impact than education and income. Another possible explanation is that unintentional bias may lead medical professionals to view low SES MA stroke patients as less likely to benefit from IRF, as seen in surgical literature where higher SES NHW patients are often perceived as more likely to benefit from intensive rehabilitation.^29^ In addition, MA patients had more severe prestroke disability than NHW patients (61% of MA had mRS 2+ compared with 54% for NHW), so perhaps intensive rehabilitation was not possible, despite having more severe strokes (median MA NIHSS=4 vs median NHW NIHSS=3).

In addition, or alternatively, perhaps cultural differences contribute to the family’s preference for rehabilitation placement. We have previously shown that MA stroke survivors receive more help from informal caregivers than NHWs despite no differences in stroke severity.^30^ Familism is an important aspect of H/L culture that emphasizes family member emotional and financial support,^31^ which may suggest a preference for home-based informal care. In this study, we found significant differences in care transitions, with MAs more likely to go home without rehabilitation and NHWs more likely to receive some rehabilitation. How familism and cultural practices are associated with SES remains to be determined.

We additionally examined transition patterns among MA and NHW stroke patients. MA stroke patients were more likely to have complex transitions between the first and second rehabilitation provider than NHW patients. The reason for this difference may lie in the choice for the first rehabilitation provider. Speculatively, if the first rehabilitation provider was inadequate, MA stroke patients may need to transition to a higher intensity provider to meet their rehabilitation needs.

This was the first population-based study looking at poststroke postacute care for MAs. Strengths of this study include the community-based nature and established infrastructure of the BASIC project in Nueces County, as we were able to recruit a large number of MA participants for this study.

### Study limitations

A limitation of this study is that the sample size was too small to conduct subgroup analyses. Furthermore, we did not include a robust analysis of the transitions between rehabilitation providers in this study, which limits our ability to draw conclusions regarding their impact on rehabilitation. In addition, in-hospital complications were determined by International Classification of Disease-10 codes, which may not be completely reliable. Complications were included in the sensitivity analysis as they might affect patients’ eligibility for IRF; however, patients who may have received lower-quality care could have been classified as more complex than they were at the start of care. We also do not have poststroke functional status data before 90 days, limiting our ability to determine how rehabilitation provider affects functional gains. Finally, this project examined a single county, so generalizations to other communities should be done with caution.

## Conclusions

In conclusion, a lower percentage of MA stroke patients went to IRF compared with NHW stroke patients in the 90 days after stroke. In this population-based study, we identified SES as the major driver of the difference. Future research might focus on the interaction between SES and race and ethnicity, including cultural issues and possible provider bias, to better understand the gaps that may drive this disparity.

## Supplementary Material

1

2

3

4

5

6

## Figures and Tables

**Table 1:** Patient demographic and clinical characteristics by rehabilitation and ethnicity.

Variable	% Missing	MAs (n=954) Median/Count (IQR/%)	NHWs (n=499) Median/Count (IQR/%)	*P* Value	Any Rehab (n=974)			
					% Missing	MAs (n=660) Median/Count (IQR/%)	NHWs (n=314) Median/Count (IQR/%)	*P* Value
Income*	9.6			<.001	10.5			<.001
<$10,000		174 (20.4)	52 (11.3)			120 (20.4)	25 (8.8)	
$10,000-$19,999		200 (23.4)	50 (10.9)			138 (23.5)	34 (11.9)	
$20,000-$29,999		140 (16.4)	79 (17.2)			108 (18.4)	50 (17.5)	
$30,000-$49,999		127 (14.9)	89 (19.4)			78 (13.3)	59 (20.7)	
$50,000+		214 (25.0)	189 (41.2)			143 (24.4)	117 (41.1)	
Education	0.6			<.001	0.4			<.001
<High school		365 (38.5)	41 (8.2)			269 (40.9)	23 (7.3)	
High school		300 (31.6)	126 (25.4)			196 (29.8)	73 (23.3)	
>High school		283 (29.9)	330 (66.4)			192 (29.2)	217 (69.3)	
Comorbidities		3 (2, 4)	3 (1, 4)	.031	0.0	3 (2, 4)	3 (2, 4)	.677
Complications	–			–				
Sepsis		–	–		0.0	20 (3.0)	13 (4.1)	.448
Falls		–	–		0.0	27 (4.1)	23 (7.3)	.042
Pneumonia		–	–		0.0	42 (6.4)	28 (8.9)	.184
Pressure ulcers		–	–		0.0	6 (0.9)	2 (0.6)	>.99
Acute kidney failure		–	–		0.0	121 (18.3)	65 (20.7)	.384
Urinary tract infection		–	–		0.0	46 (7.0)	26 (8.3)	.513
Current smoker	0.1	226 (23.7)	129 (25.8)	.403	0.1	138 (20.9)	60 (19.1)	.551
Prestroke cognition	16.0			.009	12.6			.222
IQCODE ≤3*		390 (47.8)	195 (48.1)			269 (45.7)	126 (48.1)	
IQCODE 3.01-3.43		253 (31.0)	152 (37.5)			188 (31.9)	91 (34.7)	
IQCODE 3.44+		173 (21.2)	58 (14.3)			132 (22.4)	45 (17.2)	
Prestroke disability	0.1			.005	0.2			.041
mRS 0-1		377 (39.6)	231 (46.4)			251 (38.1)	132 (42.2)	
mRS 2-3		444 (46.6)	224 (45.0)			305 (46.3)	150 (47.9)	
mRS 4-5		132 (13.9)	43 (8.6)			103 (15.6)	31 (9.9)	
Age (y)	0.0	65 (57, 73)	69 (59.5, 78)	<.001	0.0	68 (59, 77)	73 (65, 81)	.000
Male	0.0	512 (53.7)	269 (53.9)	.956	0.0	337 (51.1)	160 (51.0)	>.99
Initial NIHSS	0.6	4 (1, 7)	3 (1, 6)	.060	0.6	4 (2, 9)	4 (2, 8)	.295
History of stroke/TIA*	1.2	295 (31.4)	152 (30.7)	.811	1.3	208 (32.0)	111 (35.8)	.242
tPA/thrombectomy	1.7	169 (18.1)	123 (24.9)	.003	0.7	122 (18.7)	82 (26.2)	.009
Uninsured/Medicaid only		225 (23.8)	78 (15.7)	.003	0.6	125 (19.1)	29 (9.3)	<.001

Abbreviations: IQCODE, Informant Questionnaire on Cognitive Decline in the Elderly; IQR, interquartile range; mRS, modified Rankin score; MA, Mexican American; NHW, non-Hispanic White; NIHSS, NIH Stroke Scale; TIA; tPA, tissue plasminogen activator.

* Denotes >1% missing data.

**Table 2:** Initial rehabilitation provider after acute stroke.

Placement	At Least 2 Calls (n=1453)			Any Rehab (n=974)		
	MA (%)	NHW (%)	Total (%)	MA (%)	NHW (%)	Total (%)
IRF	23.8	26.9	24.5	35.7	43.0	38.0
SNF	10.2	7.6	9.3	15.3	14.6	15.2
HHA*	25.8	20.0	23.8	37.5	32.2	35.7
OP*	7.9	6.2	7.3	11.5	10.2	11.1
None	32.4	39.3	34.8	–	–	–

Abbreviations: HHA, home health agency; IRF, inpatient rehabilitation facility; MA, Mexican American; NHW, non-Hispanic White; OP, outpatient rehabilitation; SNF, skilled nursing facility.

* One participant’s rehab type was not listed, determined to be either HHA or OP.

**Table 3:** Counts of transitions (ethnicity table %) between the first 2 providers for MA vs NHW stroke patients, among participants with at least 2 calls (n=1453).

		Location 2					
Ethnicity	Location 1	Home	Home + HHA	Home + OP	IRF	Other	SNF
MA	Home	304 (31.9)	57 (6)	44 (4.6)	8 (0.8)	2 (0.2)	3 (0.3)
	Home + HHA	151 (15.8)	25 (2.6)	4 (0.4)	3 (0.3)	0 (0)	4 (0.4)
	Home + OP	13 (1.4)	6 (0.6)	9 (0.9)	0 (0)	0 (0)	0 (0)
	IRF	70 (7.3)	85 (8.9)	27 (2.8)	11 (1.2)	2 (0.2)	19 (2)
	Other	0 (0)	1 (0.1)	0 (0)	5 (0.5)	8 (0.8)	5 (0.5)
	SNF	25 (2.6)	33 (3.5)	1 (0.1)	9 (0.9)	2 (0.2)	18 (1.9)
NHW	Home	193 (38.7)	27 (5.4)	12 (2.4)	6 (1.2)	0 (0)	0 (0)
	Home + HHA	49 (9.8)	12 (2.4)	7 (1.4)	1 (0.2)	0 (0)	1 (0.2)
	Home + OP	12 (2.4)	2 (0.4)	3 (0.6)	0 (0)	0 (0)	0 (0)
	IRF	34 (6.8)	53 (10.6)	12 (2.4)	1 (0.2)	2 (0.4)	25 (5)
	Other	1 (0.2)	0 (0)	0 (0)	1 (0.2)	7 (1.4)	3 (0.6)
	SNF	5 (1)	11 (2.2)	1 (0.2)	4 (0.8)	1 (0.2)	13 (2.6)

Abbreviations: HHA, home health agency; IRF, inpatient rehabilitation facility; MA, Mexican American; NHW, non-Hispanic White; OP, outpatient rehabilitation; SNF, skilled nursing facility.

**Table 4:** Sequential modeling of patient demographic and clinical variables on initial rehabilitation provider, reporting the ethnicity effect and the percent change of the ethnicity effect with the addition of covariates from the unadjusted model.

Model	Ethnicity Point Difference (NHW vs MA) (%)	Lower 95% Confidence Limit (%)	Upper 95% Confidence Limit (%)	Percent Change From Model 0	*P* Value
Model 0	7.4	0.8	14	0.0	.028
Model 0 + sex	7.4	0.9	14	0.4	.027
Model 0 + age	7.0	0.3	13.7	−5.4	.040
Model 0 + NIHSS	7.9	1.5	14.4	7.3	.016
Model 0 + thrombolysis/thrombectomy	6.9	0.3	13.5	−6.1	.040
Model 0 + prestroke mRS	6.7	0.1	13.2	−9.6	.046
Model 0 + prestroke IQCODE	7.1	0.6	13.7	−3.9	.034
Model 0 + comorbidity	7.5	0.9	14.0	1.0	.027
Model 0 + smoker	7.3	0.7	13.9	−1.4	.030
Model 0 + history stroke/TIA	7.5	0.9	14.1	1.7	.025
Model 0 + education	3.4	−3.9	10.6	−54.6	.363
Model 0 + income	5.3	−1.5	12.1	−28.2	.121
Model 0 + insurance	6.6	0.0	13.2	−10.5	.051
Model 0 + hospital system	6.0	−0.6	12.6	−19.1	.077
Model 1 (Model 0 + demographics)	6.9	0.3	13.6	−6.3	.042
Model 2 (Model 1 + clinical)	6.5	−0.0	13.0	−12.0	.051
Model 3 (Model 2 + comorbidity burden)	6.4	−0.1	13.0	−12.5	.053
Model 4 (Model 3 + SES)	1.8	−5.4	8.9	−76.3	.629
Model 5 (Model 4 + insurance)	1.9	−5.2	9.0	−74.3	.602
Model 6 (Model 5 + hospital system)	1.1	−6.0	8.1	−85.6	.768

Abbreviations: IQCODE, Informant Questionnaire on Cognitive Decline in the Elderly; MA, Mexican American; mRS, modified Rankin score; NIHSS, National Institute of Health Stroke Scale; NHW, non-Hispanic White; SES, socioeconomic status; TIA.

## Data Availability

Anonymized data not published within this article will be made available by request from any qualified investigator.

## List of abbreviations:

BASIC-PAC: Brain Attack Surveillance in Corpus Christi Post Acute Care
H/L: Hispanic/Latin(a,e,o,x)
HHA: home health agency
IRF: inpatient rehabilitation facility
MA: Mexican American
mRS: modified Rankin Scale
NHW: non-Hispanic White
NIHSS: National Institute of Health Stroke Scale
PS: propensity-score
SES: socioeconomic status
SNF: skilled nursing facility
