# Supplementary material for: Ethnic Differences in Postacute Stroke Rehabilitation
Source: Arch Phys Med Rehabil. Author manuscript; Available in PMC 2026 Sep 22. (PMC13596767; doi:10.1016/j.apmr.2025.11.008)
Supplement: Supplementary file 1 [file NIHMS2209067-supplement-1.docx]

Supplemental Table S1: Patient demographic and clinical characteristics by rehabilitation and ethnicity.

|  | **IRF as 1^st^ provider** | | |
| --- | --- | --- | --- |
|  | **MAs (n=235)** | **NHWs (n=135)** |  |
| **Variable** | **Median/**  **Count (IQR/%)** | **Median/**  **Count (IQR/%)** | **P-value** |
| Income* |  |  | <0.001 |
| < $10,000 | 39 (18.7) | 8 (6.6) |  |
| $10,000-$19,999 | 44 (21.1) | 12 (9.8) |  |
| $20,000-$29,999 | 34 (16.3) | 26 (21.3) |  |
| $30,000-$49,999 | 30 (14.4) | 23 (18.9) |  |
| $50,000+ | 62 (29.7) | 53 (43.4) |  |
| Education |  |  | <0.001 |
| < High School | 81 (34.6) | 6 (4.5) |  |
| High School | 72 (30.8) | 34 (25.4) |  |
| > High School | 81 (34.6) | 94 (70.1) |  |
| Comorbidities | 3 (2, 4) | 3 (2, 4) | 0.139 |
| Complications |  |  |  |
| Sepsis | 12 (5.1) | 8 (5.9) | 0.81 |
| Falls | 13 (5.5) | 10 (7.4) | 0.506 |
| Pneumonia | 19 (8.1) | 14 (10.4) | 0.455 |
| Acute Kidney Failure | 47 (20) | 28 (20.7) | 0.894 |
| Urinary Tract Infection | 17 (7.2) | 16 (11.9) | 0.184 |
| Current Smoker | 49 (20.9) | 23 (17.0) | 0.415 |
| Pre-stroke Cognition |  |  | 0.928 |
| IQCODE <= 3* | 105 (49.3) | 57 (50.9) |  |
| IQCODE 3.01-3.43 | 77 (36.2) | 38 (33.9) |  |
| IQCODE 3.44+ | 31 (14.6) | 17 (15.2) |  |
| Pre-Stroke Disability |  |  | 0.689 |
| mRS 0-1 | 103 (43.8) | 65 (48.5) |  |
| mRS 2-3 | 108 (46.0) | 57 (42.5) |  |
| mRS 4-5 | 24 (10.2) | 12 (9.0) |  |
| Age (years) | 68 (59, 77) | 73 (66, 81) | 0.001 |
| Male | 132 (56.2) | 73 (54.1) | 0.745 |
| Initial NIHSS | 6 (3, 11) | 6 (3, 12) | 0.366 |
| History of Stroke/TIA* | 67 (29.1) | 50 (37.3) | 0.130 |
| tPA/Thrombectomy | 48 (20.7) | 40 (29.6) | 0.058 |
| Uninsured/Medicaid only | 39 (16.8) | 9 (6.7) | 0.006 |
| * Denotes >1% missing data  IQCODE = Informant Questionnaire on Cognitive Decline in the Elderly  mRS = modified Rankin score  NIHSS = NIH Stroke Scale  tPA = tissue plasminogen activator | | | |
