# Supplementary material for: Ethnic Differences in Postacute Stroke Rehabilitation
Source: Arch Phys Med Rehabil. Author manuscript; Available in PMC 2026 Sep 22. (PMC13596767; doi:10.1016/j.apmr.2025.11.008)
Supplement: Supplementary file 2 [file NIHMS2209067-supplement-2.docx]

Supplemental Table S2: Transition from lower-to-higher, higher-to-lower, or static post-acute rehabilitation provider between MA versus NHW patients.

|  | **MA** | **NHW** | **p-value < 0.001** |
| --- | --- | --- | --- |
| Higher to lower | 406 (42.8%) | 178 (35.9%) |  |
| Static | 420 (44.3%) | 271 (54.6%) |  |
| Lower to higher | 123 (13.0%) | 47 (9.5%) |  |
