# Supplementary material for: Ethnic Differences in Postacute Stroke Rehabilitation
Source: Arch Phys Med Rehabil. Author manuscript; Available in PMC 2026 Sep 22. (PMC13596767; doi:10.1016/j.apmr.2025.11.008)
Supplement: Supplementary file 3 [file NIHMS2209067-supplement-3.docx]

Supplemental Table S3: Unadjusted model versus propensity-score (PS) adjusted model accounting for confounding variables comparing the effect of ethnicity on initial rehabilitation provider.

| **Model** | **Estimate** | **P-value** |
| --- | --- | --- |
| Unadjusted, NHW vs MA | 0.074 (0.008, 0.140) | 0.028 |
| PS-adjusted, NHW vs MA | 0.016 (-0.060, 0.093) | 0.678 |
| NHW = non-Hispanic White  MA = Mexican American | | |
