# Supplementary material for: Ethnic Differences in Postacute Stroke Rehabilitation
Source: Arch Phys Med Rehabil. Author manuscript; Available in PMC 2026 Sep 22. (PMC13596767; doi:10.1016/j.apmr.2025.11.008)
Supplement: Supplementary file 4 [file NIHMS2209067-supplement-4.docx]

Supplemental Table S4: Ethnic effects by income and education on the ethnic difference in IRF utilization in an interaction model.

| Variable | Effect | LCL | UCL | Interaction p-value |
| --- | --- | --- | --- | --- |
| Education |  |  |  |  |
| NHW vs MA, < High school | -0.102 | -0.291 | 0.087 | 0.235 |
| NHW vs MA, > High school | -0.002 | -0.104 | 0.101 |  |
| NHW vs MA, High school | 0.097 | -0.043 | 0.236 |  |
| Income |  |  |  | 0.501 |
| NHW vs MA, $20,000-$29,999 | 0.157 | -0.038 | 0.352 |  |
| NHW vs MA, $30,000-$49,999 | -0.017 | -0.202 | 0.169 |  |
| NHW vs MA, $50,000+ | -0.01 | -0.136 | 0.116 |  |
| NHW vs MA, <$20,000 | -0.011 | -0.158 | 0.136 |  |
