# Supplementary material for: Ethnic Differences in Postacute Stroke Rehabilitation
Source: Arch Phys Med Rehabil. Author manuscript; Available in PMC 2026 Sep 22. (PMC13596767; doi:10.1016/j.apmr.2025.11.008)
Supplement: Supplementary file 5 [file NIHMS2209067-supplement-5.docx]

Supplemental Table S5: Sensitivity Analyses.

|  | Analysis | Risk difference | LCL | UCL | p-value |
| --- | --- | --- | --- | --- | --- |
| NHW vs MA | Propensity score with additional adjustment | 0.02 | -0.053 | 0.094 | 0.586 |
| NHW vs MA | Complications added to propensity score | 0  .012 | -0.065 | 0.088 | 0.776 |
