# Supplementary material for: Ethnic Differences in Postacute Stroke Rehabilitation
Source: Arch Phys Med Rehabil. Author manuscript; Available in PMC 2026 Sep 22. (PMC13596767; doi:10.1016/j.apmr.2025.11.008)
Supplement: Supplementary file 6 [file NIHMS2209067-supplement-6.docx]

Supplemental Table S6: Weighted Results.

|  | estimate | p-value | LCL | UCL |
| --- | --- | --- | --- | --- |
| MA vs NHW, Unadjusted | 0.078 | 0.035 | 0.008 | 0.140 |
| MA vs NHW, PS-adjusted | 0.040 | 0.363 | -0.060 | 0.093 |
